# Supplementary material for: Molecular Regulation of the RhoGAP GRAF3 and Its Capacity to Limit Blood Pressure In Vivo
Source: Cells. 2020 Apr 22;9(4):1042. doi: 10.3390/cells9041042 (PMC7226614; doi:10.3390/cells9041042)
Supplement: Supplementary file 1 [file cells-09-01042-s001.zip › cells-781590-supplementary-original/Cells_resub_Dee_etal_2020_supplemental.pdf]

cells

Supplemental Material

Molecular regulation of the RhoGAP GRAF3 and its capacity to limit blood pressure in vivo

Rachel A. Dee [1,†], Xue Bai[1,†], Christopher P. Mack [1,2] and Joan M. Taylor [1,2,*]

[1] Department of Pathology and Laboratory Medicine, University of North Carolina at Chapel Hill, Chapel Hill, NC, 27599, USA
[2] McAllister Heart Institute, University of North Carolina at Chapel Hill, Chapel Hill, NC, 27599, USA
[†] Authors contributed equally
[*] Correspondence: joan_m_taylor@med.unc.edu

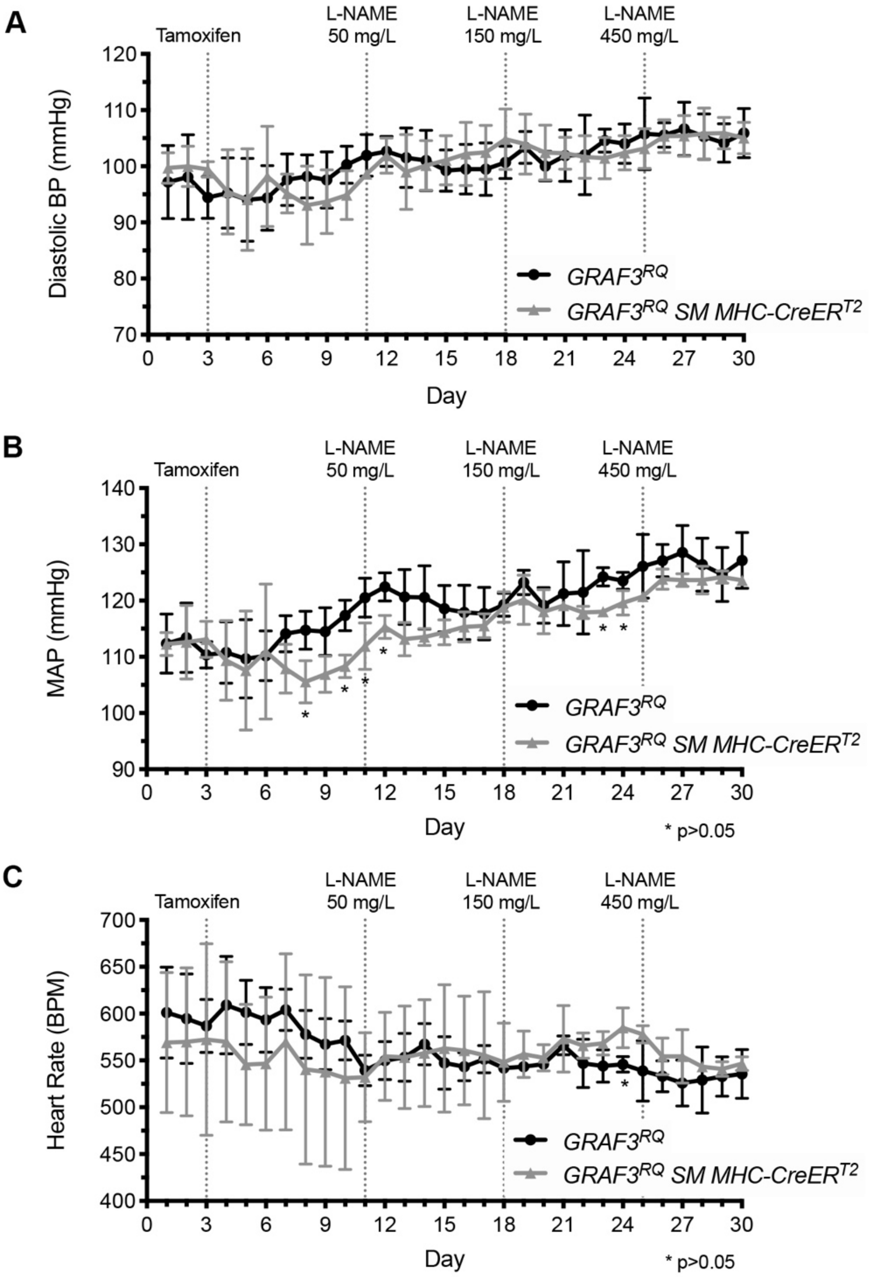

Figure S1. SMC-specific GRAF3^{RQ} expression had insignificant impact on diastolic BP, MAP, or HR. Average 24hr **(a)** diastolic BP **(b)** MAP, and **(c)** HR measured via radio-telemetry, of unrestrained, conscious *GRAF3^{RQ}* and *GRAF3^{RQ} SM MHC-CreER^{T2}* mice after tamoxifen treatment (100mg/kg for 3 consecutive days) and increasing L-NAME doses (50mg/L, 150mg/L, or 450mg/L) given for a week (each) in drinking water. Data are expressed as mean ± SD; n=4 for *GRAF3^{RQ}* mice and n=3 for *GRAF3^{RQ} SM MHC-CreER^{T2}*.*p>0.05 vs.GRAF3^{RQ} (Student's t-test).

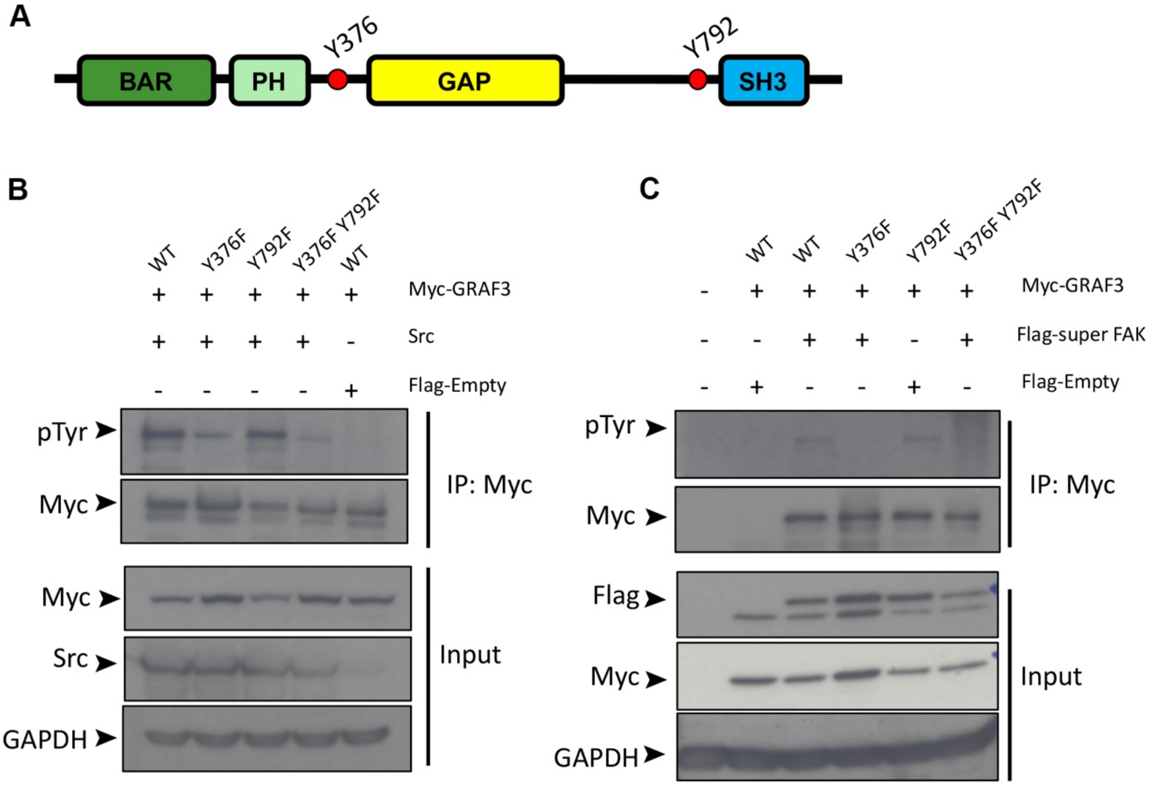

Figure S2. Y792 is not a major Src or FAK phosphorylation site. **(a)** Schematic of GRAF3 domain structure with indicated location of Y376 and Y792. Cos cells were transfected with the indicated GRAF3 variant and either **(b)** ^{529F}Src or **(c)** superFAK. Myc-tagged GRAF3 was immunoprecipitated from cell lysate and probed for tyrosine phosphorylation.

Video S1. Predicted structure of the open (active) GRAF3 BAR-PH-GAP dimer. Color scheme follows: BAR 1 (dark purple), PH 1 (light purple), BAR 2 (dark green), PH 2 (light green), GAP 1 and 2 (yellow), arginine fingers (active site) (dark blue), RhoA docking sites (pink), C-terminus of PH domain (red), N-terminus of GAP domain (orange); residues in teal aids in visualizing rotation. Model was created using Pymol and the solved, similar structures of Appl1 (BAR-PH) and GRAF1 (GAP).
